# Using a short‐term risk assessment and compassion focused staff support groups to reduce restrictive intervention use in a secure mental health service

**DOI:** 10.1111/bjc.70004

**Published:** 2025-07-06

**Authors:** Daniel Lawrence, Daniel Stubbings, Andrew Watt

**Affiliations:** ^1^ NHS Wales Cardiff UK; ^2^ Department of Applied Psychology Cardiff Metropolitan University Cardiff UK

**Keywords:** compassion, DASA, forensic psychiatry, maintenance model of restrictive practices, restrictive practice

## Abstract

**Objectives:**

The aim of the current study was to introduce two interventions to reduce restrictive practice use in a UK‐based secure mental health service. The interventions were a short‐term risk assessment called the Dynamic Appraisal of Situational Aggression (DASA), and staff support groups based on the Compassion Focused Therapy model (CFSS groups). Intervention selection was guided by a recently published, trauma‐informed model of restrictive practice use, the Maintenance Model of Restrictive Practices.

**Methods:**

Five secure mental health wards were included in the study and restrictive practices were compared pre and post intervention for each ward. Owing to limitations in the available data, we were limited in the research design and analyses that could be used. Descriptive statistics were used to compare restraint frequency and short‐, medium‐ and long‐duration seclusion episodes pre and post intervention, per ward.

**Results:**

DASA was associated with some reductions in restrictive intervention use on some wards, but this was inconsistent. Similarly, CFSS groups were associated with some limited reductions in restrictive intervention use, but this was again inconsistent. When the DASA was introduced in addition to CFSS groups, reductions in physical restraints and medium and long‐duration seclusion episodes were observed.

**Conclusions:**

Short‐term risk assessment and compassion focused staff groups were associated with reductions in restrictive practice use across some secure mental health wards, but this was inconsistent. To our knowledge, this is the first study that has addressed staff emotional wellbeing in an attempt to reduce restrictive practice use. The findings provide some tentative support for the Maintenance Model of Restrictive Practices.

## INTRODUCTION

Restrictive practices in care settings (also referred to as restrictive interventions; coercion; coercive measures; and containment) are intentional interventions by staff that restrict a service user's movement, liberty and/or freedom. At their most extreme, restrictive practices include physical restraint and seclusion (Department of Health (DoH), 2014). Restrictive practices are meant to be a last resort because their use has been associated with harmful effects for service users. Harmful effects of restrictive practices include physical health problems, physical injury, deterioration of mental health (including the onset of post‐traumatic stress disorder), increased length of stay in hospital (Chieze et al., 2019) and, in extreme instances, death (Duxbury et al., 2011; Weiss et al., 1998). Substantial effort has been made to reduce restrictive practice use across inpatient mental health services, but they remain a prevalent international problem (O'Donovan et al., 2022).

Lawrence et al. (2024) argued that the lack of progress with reducing restrictive practices was in part due to limitations associated with existing theoretical models. The authors observed that existing models typically provided guidance for intervention but often without an explanatory framework; they were limited in their ability to explain the core factors that lead to restrictive practices being initiated and maintained. Furthermore, interventions arising from these models were intended to be implemented in the same way across all services. Lawrence et al. (2024) stressed that existing models fail to account for complex interactions between factors that result in restrictive practices being applied differently in different types of services (Goodwin et al., 2024; Lawrence, Davies, et al., 2025). As an alternative, Lawrence and colleagues developed the Maintenance Model of Restrictive Practices (MMRP) which has the potential for application across all areas of mental health practice where restrictive practices are used. The MMRP is an integrated theoretical model that explains the (repeated) use of restrictive practices in inpatient mental health settings. The MMRP was designed to provide a framework that would allow service providers and clinicians to understand the core factors that drive restrictive practice use in their specific services, so they could then act to reduce restrictive practices by targeting relevant factors through intervention. The aim of the current study was to test this model by introducing two interventions that aimed to directly target two of the MMRP components, namely service user risk behaviour and staff's ability to manage their threat‐based emotional responding.

Risk assessment tools aim to assist with the identification and management of individuals at risk of harmful behaviour (Singh et al., 2011). They are useful because they aid the decision‐making of clinicians. Unstructured decision‐making regarding risk behaviours has limitations (Fogel, 2009) not least because clinicians are prone to heuristic bias, often resulting in an overestimation of risk (Daffern & Ogloff, 2019). This is one consequence of working in an uncertain environment where risk incidents are frequent but difficult to predict. These conditions mean that threat systems of mental health staff can be oversensitive and result in restrictive practices being used when a threat is anticipated but not actually present. Risk assessments can protect against this problem by facilitating more objective and accurate identification of risk behaviours (Griffith et al., 2013). They also provide the opportunity to identify potential risk incidents early so that proactive interventions can be employed before a situation escalates to the point where an incident takes place or restrictive practices are required (Maguire et al., 2017). This idea has been supported by the empirical evidence for short‐term risk assessments (Abderhalden et al., 2008; Maguire et al., 2019; van de Sande et al., 2011).

Responding to service user behaviour with anxiety or anger, by the professionals involved in their care, is unsurprisingly common given the prevalence of aggression displayed on mental health wards (Pulsford et al., 2013). The capacity for staff to become traumatized by their work, both directly (Newman et al., 2021) and vicariously (Molnar et al., 2017) is well established. Lucre and Taylor (2020) acknowledged this and summarized that there is a ‘cost to caring’ for professionals who provide support for forensic service users. This is problematic from a staff well‐being perspective, but also because threat‐based emotions such as anger (Jalil et al., 2017) and fear (Bowers et al., 2007), as well as low morale (Hui et al., 2016), have been linked to increased restrictive practice use. Excessive restrictive practice use is associated with unhelpful coping mechanisms employed by staff (Hui et al., 2016) that can block their capacity to provide compassionate high‐quality care (Bowers et al., 2011). In other studies, staff reported worrying that restricting their ability to apply restrictive practices would increase risk of harm (Ashcraft et al., 2012) and made them fearful about the prospect of eliminating restrictive practice use (Muir‐Cochrane et al., 2018). Curran (2007) argued that actual or perceived lack of safety among staff may play a central role in their hesitance to move towards a seclusion/restraint free environment.

Cognitive strategies such as detachment that staff employ to manage their emotional distress can increase the risk of them ‘projecting’ unwanted feelings onto service users and treating them with contempt or disgust, and with reduced compassion (Lucre & Taylor, 2020). It is reasonable to argue that such psychological processes could facilitate greater restrictive practice use by staff who feel this way. These processes have implications for staffs' attitudes and motivation to engage with reducing restrictive practice interventions and explain why some authors have reported that the ‘pull’ of restrictive practices is powerful, even when interventions have been able to reduce them (Duxbury et al., 2019).

Compassion Focused Therapy (CFT; Gilbert, 2009) is a psychological model that aims to support individuals to notice and understand how biopsychosocial motivations influence their behaviour and increase the likelihood of them behaving in harmful ways. It has been argued that CFT can help people develop the intention and capacity to stimulate affiliative emotional systems and behave more compassionately towards themselves and others. Gilbert (2014) argues that these capacities are central to self‐regulation, well‐being and prosocial behaviour. Of relevance to the current research is that improved emotion regulation is a central therapeutic focus of CFT, which Irons (2022) describes as the process of alleviating people from their distress associated with feeling overwhelmed by their emotional experiences. CFT has been found to be effective for improving emotional regulation and reducing distress in both clinical samples (Craig et al., 2020), as well as for people in the general population (Irons & Heriot‐Maitland, 2021). Staff support groups based on the CFT model were introduced as an intervention for this reason and to target the staff threat component of the MMRP.

The aim of this study was to explore the effect of two interventions in a secure mental health service on rates of restrictive practice use. We were interested in extreme forms of restrictive practices (often termed restrictive interventions) namely seclusion and physical restraint. The interventions introduced were the Dynamic Appraisal of Situational Aggression (DASA) short‐term risk assessment tool and Compassion Focused Staff Support (CFSS) groups. To the knowledge of the author, no previous studies have considered the use of staff emotional support groups for the purpose of reducing restrictive practices. By introducing such alongside an already established approach such as short‐term risk assessment, it was possible to consider the effect each intervention had on wards, but it also allowed exploration of whether combining the two interventions on the same ward had any additional benefit. Based on the MMRP and its main hypothesis, the current study aimed to test the following predictions:

- Implementation of the DASA would lead to reduced restrictive intervention use.
- Implementation of CFSS groups would lead to reduced restrictive intervention use.
- Implementation of the DASA as well as CFSS groups would result in additional improvements in restrictive intervention use compared to just CFSS groups alone.

## METHOD

### Design

It had originally been planned that a quasi‐experimental, pre/post design be implemented for this study. Independent variables would have been the interventions, namely implementation of the DASA risk assessment tool and CFSS groups. Dependent variables would have been restrictive intervention use, specifically frequency of physical restraints, frequency of seclusion and duration of seclusion episodes. The study would have consisted of three conditions: (1) DASA only, implemented on three wards; (2) CFSS group only (two wards); and (3) CFSS group and DASA combined (one ward). However, the data available for analysis was limited as it was routinely recorded at ward level, and restrictive interventions could not therefore be related to individual patients, and the independence of observations in the various pre and post intervention conditions could not be assured. This limitation made the use of inferential statistical analysis unviable; we therefore opted to report a series of ward‐level case studies, where pre and post levels of restrictive interventions were reported descriptively. Additionally, it was originally planned that we would report psychometric outcomes related to staff attitudes towards the use of restrictive practices. However, it was only possible to collect such data across two of the five wards, and as we were not able to report this for all conditions of the study, the decision was made not to report this outcome.

### Research site and sample

The research took place in a secure hospital in the United Kingdom, which provided care and treatment to service users in medium (MSU) and low secure wards (LSU). Service users at the site consisted of adult men and women predominantly of working age (18–65 years old). The hospital consisted of 96 beds split across seven wards. The DASA was introduced on three wards, and the CFSS groups were introduced on two wards. On one of the wards, the DASA was introduced approximately 6 months after the CFSS groups had started. See Table 1 for an overview of wards included in the study. It had initially been planned that another ward would be involved in the study, another low secure ward for adult men. However, due to resource issues, it was not possible to implement either intervention on this ward and it was therefore not included. At the time of the research, all service users were detained under the UK Mental Health Act (1983). Charge nurses on the wards were responsible for the completion, recording and communication of DASAs each day. Those who attended the CFSS groups were ward‐based nursing staff, typically health care assistants and occasionally qualified mental health nurses.

**TABLE 1:** Wards Included in the Study.

Ward	Type	No. beds	No. staff	Intervention
1	LSU Male Neurodevelopmental	11	5 by day 3 by night	DASA
2	MSU Male	17	7 by day 5 by night	DASA
3	LSU Male	11	5 by day 3 by night	DASA
4	MSU Female	16	6 by day 5 by night	CFSS
5	MSU Male High Dependency	12	6 by day 5 by night	CFSS & DASA

### Materials and interventions

#### DASA

The DASA (Daffern & Ogloff, 2019) is a seven‐item risk assessment based on the Structured Professional Judgement model. The assessment is concerned with the likelihood of aggression being displayed by an individual in a psychiatric environment over the coming 24‐h period. Assessors are required to rate whether each of the seven risk items (e.g., irritability) were present over the previous 24 h. Items are rated on a two‐point scale (‘0’ for absent or ‘1’ for present) and then total scores calculated. Total scores can range between 0 and 7 where a score of 0 would mean that the evaluee would be considered to be ‘low’ risk of aggression over the following 24 h. Scores between 1 and 3 equate to moderate risk of aggression, and 4 and 7 high risk of aggression. The DASA is based on the Norwegian Brøset‐Violence‐Checklist (BVC; Almvik & Woods, 1998). Since its development in 2006 the DASA has been validated in various clinical settings across the world. It has repeatedly exhibited good‐to‐excellent predictive accuracy for aggression, with good internal consistency and interrater reliability (Daffern & Ogloff, 2019).

#### CFSS groups

This intervention was based on the CFT model (Gilbert, 2014) and implemented in the manner outlined by Lucre and Taylor (2020). The group work aspect consisted of five sessions facilitated by two qualified forensic psychologists, who were trained in the CFT and CFSS models. Sessions consisted of experiential practices such as breathing and imagery exercises, learning of core CFT concepts and principles and group members practicing the application of these principles to themselves personally, as well as in their work with service users. Ideally, all participants would have attended all five sessions, but due to challenges associated with nursing staff being able to consistently attend the group sessions, the manual was adapted with the intention that each session could be beneficial for participants as a standalone session. This has been applied successfully in other areas of CFT practice (Heriot‐Maitland et al., 2014). Table 2 provides an overview of the CFSS intervention (see Lucre & Taylor, 2020 or Gilbert, 2014 for detail regarding the core CFT concepts). Sessions each lasted for 1 h and were offered on a weekly basis.

**TABLE 2:** Overview of CFSS Intervention.

Session number	Topic	Content
1	Introduction and ‘tricky brains’	Safe space agreement/group contract. Introduce soothing rhythm breathing exercise. Introduce evolutionary psychology ideas and CFT concept of old and new brains. Emotion‐thought‐emotion feedback loops. Self‐criticism as an underlying process that maintains loops. Exercise—how do the loops show up in you?
2	Three circles	Soothing rhythm breathing and check in. Revisit safe space agreement. Description of the three circles—threat, drive and soothe. Motivation and emotion regulation. Three circles check in. Exercise—relating three circles to self/team/service users.
3	Compassionate kit bag	Soothing rhythm breathing and check in. Revisit safe space agreement. Importance of engaging all the senses. Description of ward ‘compassionate kit bag’ and exploring what could be included. Exercise—calm place imagery.
4	Compassion, blocks and flows	Soothing rhythm breathing and check in. Revisit safe space agreement. Definition of compassion—what it is and what it isn't. Three flows of compassion. Fears, blocks and resistances to compassion. Exercise—compassionate other imagery.
5	Compassionate self and compassionate letter writing	Soothing rhythm breathing and check in. Revisit safe space agreement. Exercise—compassionate self‐imagery. Exercise—applying qualities from imagery exercise to write a compassionate letter to themselves or another person, including a service user (n.b. this would not be shared with the service user).

#### Restrictive intervention data

Restraint and seclusion data was obtained from records held at the research site. Restraint and seclusion incidents were recorded centrally on a monthly basis, on a Microsoft Excel database. The authors accessed these databases that provided a month‐by‐month breakdown of the frequency of physical restraints, frequency of seclusion use and duration of each seclusion episode per ward.

### Procedure

Following ethical approval (NHS Research Ethics Committee) and approval at site, the first step was to provide training on how to complete and interpret the DASA for the members of staff responsible for completing the DASAs each day. In line with guidance in the DASA manual, these would be nurses in charge of running the shifts. Training was provided by members of the psychology department at the research site. DASAs were then implemented and completed every 24 h at the beginning of the day shift. They were first implemented in April 2021 and continued to be used at the research site thereafter. On a daily basis, nurses would complete a DASA for each individual service user, provide an overall score and risk rating, then communicate this to the ward manager. The ward manager would then discuss moderate and high DASA scores during the morning handover meeting with the senior management team and discuss plans for reducing or preventing risk so that it did not escalate to the point where someone was at risk of harm and requiring restrictive interventions. The nursing team were then responsible for implementing the plans decided upon. For 12 weeks following the introduction, the members of the psychology department who had provided the training provided further support sessions for the nursing staff in DASA completion and completed a number of audits for the purpose of quality assurance.

For the CFSS groups, the lead author and another registered forensic psychologist initially provided a training session on the CFT model for the relevant staff. Both facilitators had been trained in the CFT and CFSS models. The training lasted roughly 2 h and covered the basic theory and core principles of CFT as outlined by Gilbert (2014). The same facilitators then ran the CFSS groups on the relevant wards. The facilitators received monthly supervision from the lead for CFSS from the Compassionate Mind Foundation.

CFSS sessions began in April 2022. Sessions were held separately for the two wards on a weekly basis for 1 h at a time. Two to three staff members attended each session. Sessions focused on the content described in Table 2 with a particular emphasis on experiential practice and applying the CFT principles to the topics and challenges that the group members brought. Due to staffing challenges (issues with shift patterns, staff shortages, etc.), it was difficult to run sessions in a continuous and consecutive fashion. Often, they were facilitated as standalone sessions. Sessions were attended primarily by unqualified nursing staff. In total, 36 different members of staff attended the CFSS groups. Eight members of staff (22.2%) attended all five sessions and 17 (47.2%) attended at least half of them. CFSS was introduced as part of a broader effort to implement the CFT model on these wards. As well as the CFSS groups, CFT was the main treatment model used by the psychologists when working with service users. CFT‐based formulations were also used when working with multidisciplinary teams and nursing staff to understand and conceptualize service user difficulties.

Following the termination of the CFSS intervention, the DASA was introduced to one of the CFSS wards in January 2023. The above outlined DASA procedure was followed for this process.

### Statistical analysis

We had initially intended to use inferential statistics to analyse the data collected. However, specific service user information was not contained in the available data, and restraints and seclusions had been recorded per ward. Therefore, possible statistical analyses were limited and have been confined hereafter to descriptive presentation of levels of restrictive interventions.

## RESULTS

### Frequency of restraints

**FIGURE 1:**
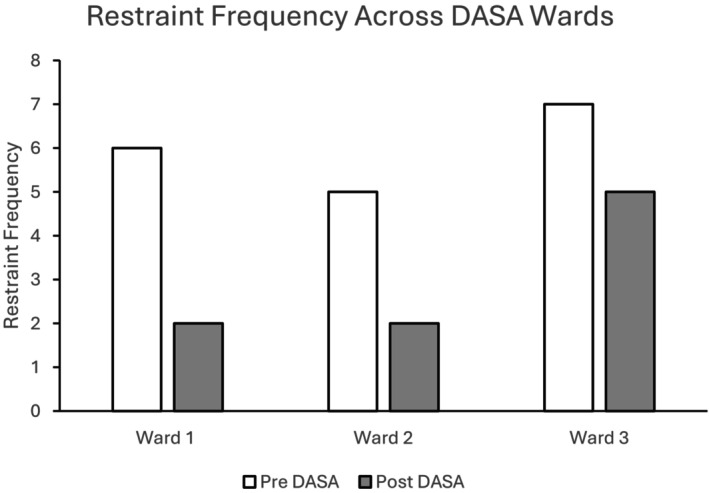
Frequency of restraints at baseline (Pre DASA) and following implementation of the DASA‐only intervention on Wards 1, 2 and 3. Ward 1 accommodated 11 low secure male service users with neurodevelopmental disabilities. Ward 2 accommodated 17 male medium secure service users. Ward 3 accommodated 11 low secure male service users.

**FIGURE 2:**
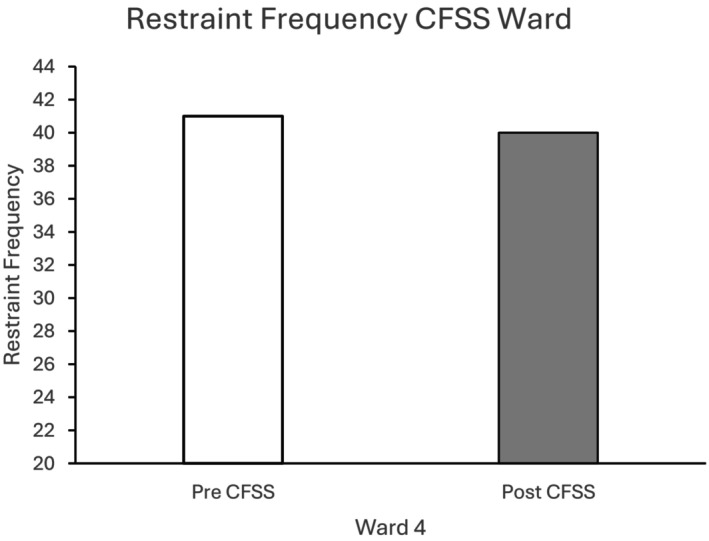
Frequency of restraints at baseline (Pre CFSS) and following implementation of the CFSS‐only intervention on Ward 4. Ward 4 accommodated 16 female service users being treated in medium security.

**FIGURE 3:**
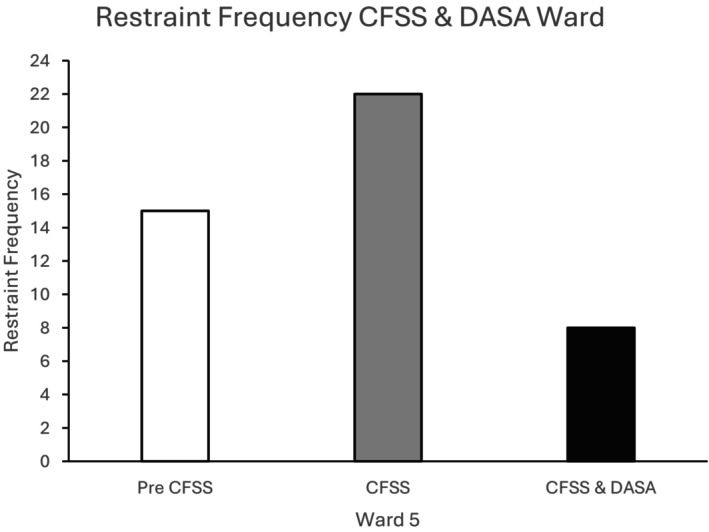
Frequency of restraints at baseline (Pre CFSS) and following implementation of the CFSS‐alone and CFSS+DASA interventions. Ward 5 accommodated 12 high dependency service users in medium security.

Physical restraint frequencies pre and post intervention per ward are provided in Figures 1, 2, 3. The figures provide restraint frequency data for 6 months pre intervention and for 6 months post intervention. Figure 1 indicates that there were reductions in the frequency of restraints in the 6 months following DASA implementation across all three wards where this intervention was introduced.

Figure 2 shows that there was a reduction in the frequency of physical restraints in the 6 months following implementation of the CFSS groups on Ward 4. This reduction was, however, minimal, where there was only one less restraint recorded in the 6 months following the intervention.

Figure 3 indicates that there was an initial increase in physical restraints on Ward 5 following the introduction of the CFSS groups. However, there was then a large reduction in physical restraints following the introduction of the DASA to a level that was lower than the recorded number of restraints at baseline.

### Duration and frequency of seclusions

**FIGURE 4:**
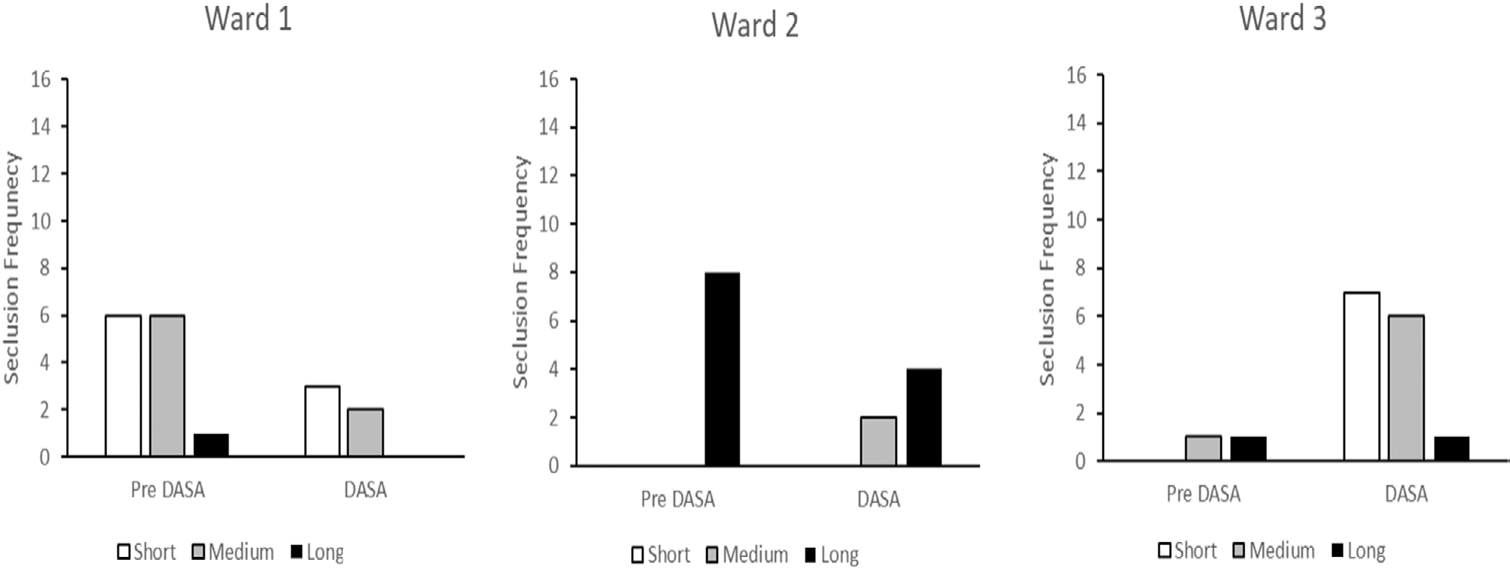
Frequency of short‐, medium‐ and long‐duration seclusions at baseline (Pre DASA) and during the implementation of the DASA‐only intervention on Wards 1, 2 and 3. Ward 1 accommodated 11 low secure male service users with neurodevelopmental disabilities. Ward 2 accommodated 17 male medium secure service users. Ward 3 accommodated 11 low secure male service users.

**FIGURE 5:**
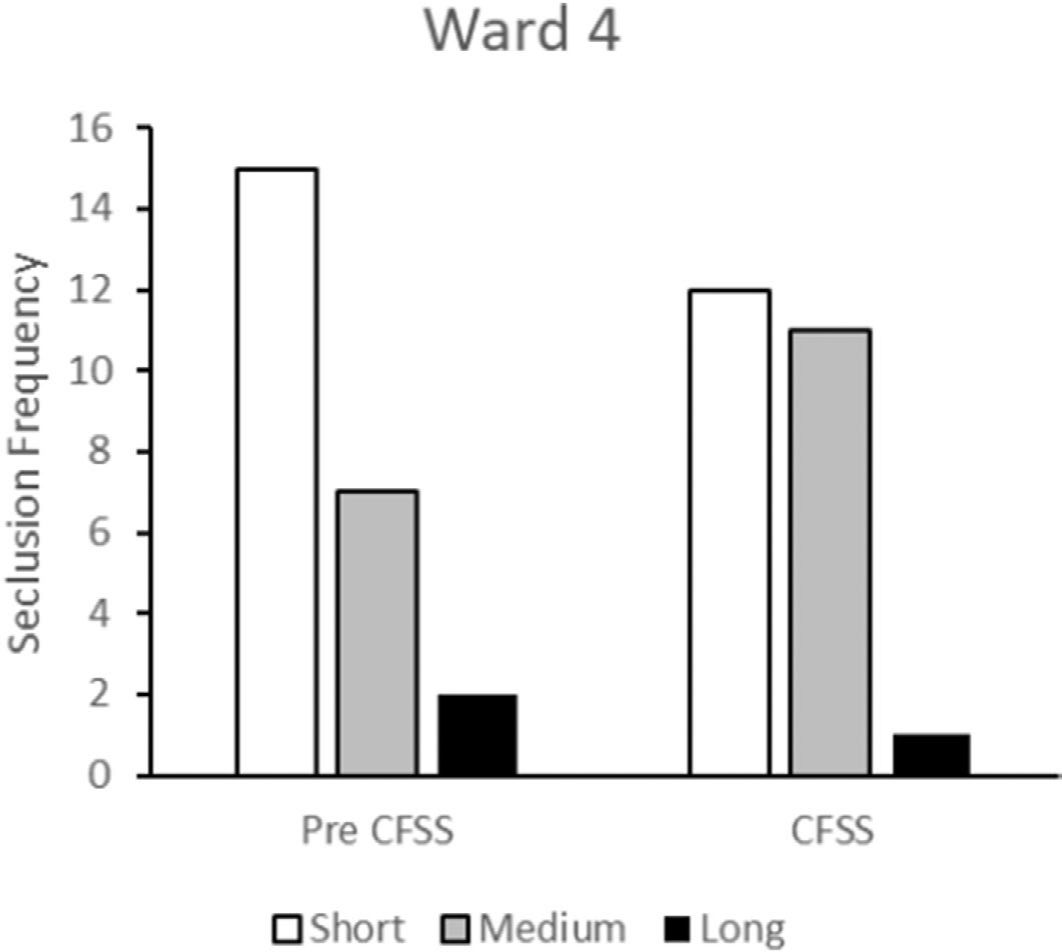
Frequency of short‐, medium‐ and long‐duration seclusions at baseline (Pre CFSS) and during implementation of the DFSS‐only intervention. Ward 4 accommodated 16 female service users being treated in medium security.

**FIGURE 6:**
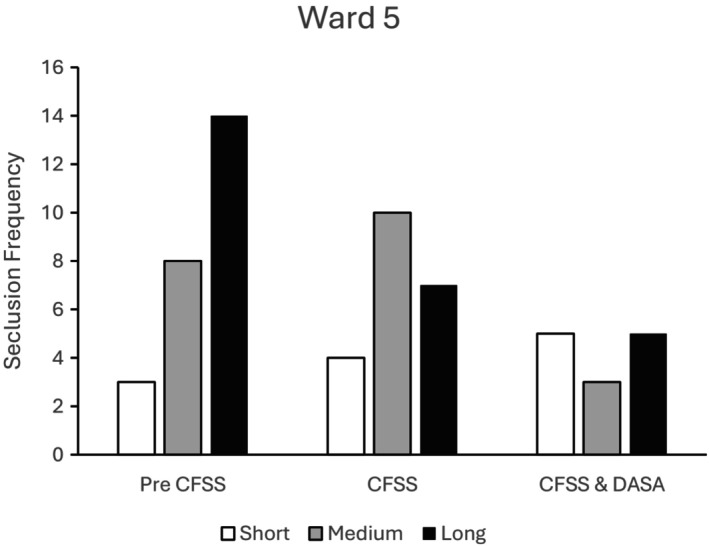
Frequency of short‐, medium‐ and long‐duration seclusions at baseline (Pre CFSS) and during implementation of the CFSS‐alone and CFSS+DASA interventions. Ward 5 accommodated 12 high dependency service users in medium security.

The average duration of seclusions was 141 h, but this varied widely; with a standard deviation of 279 h and a range between 1 and 1824 h (76 days). It was possible that unusually long or short seclusions used repeatedly with individual service users might distort the presentation of seclusion durations. Seclusion durations were therefore categorized into short‐, medium‐ and long‐duration episodes; this was achieved by rank ordering all of the seclusion durations and categorizing the shortest 33% as the short seclusions, the intermediate 33% as medium and the longest 33% as long seclusions. Resulting short seclusions ranged between 1 h and 15 h 59 min, medium seclusions ranged between 16 and 96 h, whilst long seclusions ranged from 101 to 1824 h (76 days). Categorizing the seclusion duration data in this way avoided distortion by patterns of seclusion use associated with individual (potentially atypical) service users but still permitted examination of potential changes in seclusion durations between the various conditions of the current observation. The resulting findings are presented in Figures 4, 5, 6. It is apparent from Figure 4 that there was a reduction in short‐, medium‐ and long‐duration seclusions following the introduction of the DASA on Ward 1. Following the introduction of the DASA on Ward 2, there was a 50% reduction in long‐duration seclusion episodes. There was however an increase in medium duration seclusions post‐DASA implementation. On Ward 3, however, there was an increase in both short‐ and medium‐duration seclusion episodes in the 6 months following DASA implementation. There was no change to the number of long‐duration seclusions on Ward 3.

Figure 5 presents seclusion duration frequencies for Ward 4 where the CFSS‐alone intervention was implemented. There was a reduction in the use of both short‐ and long‐duration seclusions following intervention. There was, however, an increase in medium duration seclusion episodes.

Figure 6 presents seclusion duration frequencies for Ward 5 where the CFSS and then CFSS+DASA interventions were implemented. There was a steady reduction in long‐duration seclusion episodes at each phase of the study. Medium duration seclusions increased following the introduction of the CFSS intervention but then decreased to their lowest level overall following the introduction of the DASA. The frequency of short‐duration seclusion episodes increased steadily across each phase of the study.

## DISCUSSION

The aim of this study was to begin to test the MMRP, to explore whether addressing the model components through intervention would lead to a reduction in restrictive intervention use. Due to substantial limitations regarding the data that was available, we were unable to complete the analyses originally planned, meaning that we were limited in our ability to test the predictions set out at the beginning of this manuscript. That being said, the analyses we did complete indicated that the introduction of the DASA was associated with a reduction in restrictive intervention use across some of the wards included in the study. These findings provide support to previous studies that found reductions in restrictive intervention use following the introduction of short‐term risk assessment (Abderhalden et al., 2008; Maguire et al., 2019). These effects may have been observed due to good predictive validity of the DASA (Daffern & Ogloff, 2019) which would have enabled staff to more accurately predict the likelihood of a violent incident occurring. Being able to confidently predict behavioural incidents avoids overestimation of risk and thus affords nursing staff the confidence to take more positive risks with service users which (in this case) would involve terminating seclusion earlier, thus reducing the duration of its use. The DASA was introduced to target the component of the MMRP concerned with the role of troubled or troubling behaviour in the use of restrictive practice use. The findings provide some albeit modest support for this component of the MMRP in that some improvements in restrictive practice use were observed by introducing an intervention that aimed to address it. Further, more methodologically robust research is required before this can be stated with confidence. Owing to the limitations associated with the design of the research and analyses, it is currently unclear why the DASA was not associated with reductions in restrictive intervention use across all wards. One explanation could be that, according to the authors, the MMRP is intended to be applied to services as a framework to guide intervention based on the specific ‘needs’ of the service. This is because restrictive practice use has been found to differ between different types of services and the different risks that require management (Lawrence, Davies, et al., 2025). The MMRP hypothesizes that if a service has no significant problems related to a particular component of the model, addressing it through intervention is unlikely to result in restrictive practice reductions (Lawrence et al., 2024). In the current study it was not comprehensively assessed as to whether the wards included were using restrictive interventions as a result of a specific problem that the DASA would have helped to address. This may help to explain why the DASA did not consistently result in reductions in restrictive intervention use.

Similar, inconsistent findings were observed following the introduction of the CFSS groups and to the knowledge of the author, this was the first study to investigate whether improving staff emotional regulation skills can be linked to reductions in restrictive practice use. There were, albeit limited, reductions in some areas of restrictive intervention use following the introduction of CFSS. The CFSS groups were introduced to address the component of the model concerned with staff feelings of threat‐based emotions. The aim of CFSS groups was to provide staff with emotional support and help them develop skills to regulate their emotions in response to threat and create a sense of inner safeness (Lucre & Taylor, 2020). Restrictive intervention reduction was however inconsistent and their use increased in some areas following the introduction of the CFSS groups. The reason for this inconsistency is again unclear but the explanation provided above regarding the intended application of the MMRP may provide some level of explanation.

The introduction of interventions was associated with greater reductions in restrictive practice use on the ward where they were both introduced. Reductions were observed in physical restraint use, and both medium and long duration seclusion episodes. The results again provide support for the MMRP and the overarching hypothesis that addressing multiple components of the model was associated with further improvements to restrictive intervention use than just addressing one of them. These results were observed on the high dependency male medium secure ward at the service, which provides care for a high risk, high acuity group of people. Notwithstanding the study limitations, the findings are encouraging because it has previously been found that the most well established reducing restrictive practice intervention, that is, Safewards has not been effective for reducing such practices in services for these individuals (Price et al., 2016). This could indicate that the approach to reducing restrictive practices introduced as part of the current study provides a more viable option for secure services than previous intervention models. Further research is however required to support these claims. It should also be noted that the standard Safewards model has been recognized as limited in its ability to result in changes to restrictive practice use in secure services and is currently in the process of being adapted to be made more suitable for such services (Maguire et al., 2022).

Overall, the findings provide preliminary support for the MMRP, where targeting the components of the model through the introduction of two interventions was associated with some reductions in restrictive practice use. Additionally, further improvements were observed when more than one of the model components was targeted. Whilst there was no change to some aspects of restrictive practice use in some conditions, and restrictive intervention use increased in others, this would be expected based on hypotheses derived from the MMRP model. As well as what has already been discussed about the MMRP in the previous paragraphs, restrictive practice use is a complex, multi‐faceted phenomenon (Lawrence et al., 2022) and the more components of the model addressed through intervention, the greater the reductions in restrictive practice observed (Lawrence et al., 2024). As the current study only directly addressed one or two components of the MMRP, and the ‘needs’ of the wards were not comprehensively considered beforehand, it is unsurprising that the results were inconsistent.

### Limitations

The study has several substantial limitations, meaning that the findings reported are modest and need to be interpreted with caution, and further, more methodologically robust research is required to support the claims made. One limitation was the quality of the data available. Cases were missing, and for certain types of restrictive interventions, it was only possible to calculate total frequencies per ward as opposed to restrictive intervention use per case. This limited the types of analyses that were possible. Such problems are not uncommon when using clinical data (Ibrahim et al., 2012) but support a recommendation for improvements in the way in which restrictive practice data is recorded. For example, the introduction of data recording protocols, underpinned by evidence‐based standards, ensuring seclusion episodes are accurately linked to service users through their electronic patient records, and the monitoring of staff compliance in capturing this data could be helpful.

We primarily focused on the restrictive interventions of seclusion and restraint, where there was no consideration of how the interventions introduced impacted on other restrictive interventions (e.g., enforced medication) nor on broader, lower‐level restrictive practices such as blanket restrictions or restrictive ward rules and policies. This would have been beneficial to consider because these types of restrictions have received relatively little research attention. Not only this, it has been reported that when services remove one type of restrictive practice, there is a risk that other types increase to compensate for such (Bowers et al., 2017). Considering restrictive practices beyond seclusion and physical restraint in the current study would have made it possible to control for this effect and reduce its potential bias on the results reported.

A further limitation is the lack of a randomized study design or control condition. This meant that the confidence with which the findings can be linked to the interventions introduced is limited. Results could have been influenced by several factors that were unaccounted for, including changes in the service user group and their mental health and behaviour, changes to staffing, changes to organizational leadership or policy, all of which have been linked to restrictive practice use (Lawrence et al., 2024; Lawrence, Stubbings, et al., 2025). The lack of follow‐up is another limitation, meaning that it has not been possible to comment on whether the improvements observed were sustained over the long term. It is noteworthy that some of the limitations noted in this section are related to well‐known feasibility issues associated with conducting interventions and evaluation in inpatient contexts. It is entirely possible that completing a pilot/feasibility study prior to the current study may have allowed for us to address these issues beforehand, enabling us to enhance both the interventions introduced and the evaluation of them.

## AUTHOR CONTRIBUTIONS

**Daniel Lawrence:** Conceptualization; investigation; writing – original draft; methodology; writing – review and editing; formal analysis; project administration; resources. **Daniel Stubbings:** Conceptualization; methodology; writing – review and editing; supervision. **Andrew Watt:** Conceptualization; methodology; writing – review and editing; formal analysis; supervision.

## CONFLICT OF INTEREST STATEMENT

The authors have no conflicts of interest to declare.

## Data Availability

Research data are not shared.
